# A multidisciplinary evidence-based guideline for minimally invasive surgery. Part 1: entry techniques and the pneumoperitoneum

**DOI:** 10.1007/s10397-012-0731-y

**Published:** 2012-02-22

**Authors:** Claire F. la Chapelle, Willem A. Bemelman, Bart M. P. Rademaker, Teus A. van Barneveld, Frank Willem Jansen, on behalf of the Dutch Multidisciplinary Guideline Development Group Minimally Invasive Surgery

**Affiliations:** 1 Department of Gynecology, Leiden University Medical Center, K6 room 76, P.O. Box 9600, 2300 RC Leiden, the Netherlands; 2 Department of Surgery, Academic Medical Center, Amsterdam, the Netherlands; 3 Department of Anesthesiology, Onze Lieve Vrouwe Gasthuis, Amsterdam, the Netherlands; 4 Department of Quality in Healthcare, Dutch Association of Medical Specialists, Utrecht, the Netherlands

**Keywords:** Guideline, Minimally invasive surgery, Multidisciplinary, Laparoscopy, Access, Entry, Pneumoperitoneum

## Abstract

The Dutch Society for Endoscopic Surgery together with the Dutch Society of Obstetrics and Gynecology initiated a multidisciplinary working group to develop a guideline on minimally invasive surgery to formulate multidisciplinary agreements for minimally invasive surgery aiming towards better patient care and safety. The guideline development group consisted of general surgeons, gynecologists, an anesthesiologist, and urologist authorized by their scientific professional association. Two advisors in evidence-based guideline development supported the group. The guideline was developed using the “Appraisal of Guidelines for Research and Evaluation” instrument. Clinically important aspects were identified and discussed. The best available evidence on these aspects was gathered by systematic review. Recommendations for clinical practice were formulated based on the evidence and a consensus of expert opinion. The guideline was externally reviewed by members of the participating scientific associations and their feedback was integrated. Identified important topics were: laparoscopic entry techniques, intra-abdominal pressure, trocar use, electrosurgical techniques, prevention of trocar site herniation, patient positioning, anesthesiology, perioperative care, patient information, multidisciplinary user consultation, and complication registration. The text of each topic contains an introduction with an explanation of the problem and a summary of the current literature. Each topic was discussed, considerations were evaluated and recommendations were formulated. The development of a guideline on a multidisciplinary level facilitated a broad and rich discussion, which resulted in a very complete and implementable guideline.

## Introduction

Since the early 1990s, “minimally invasive surgery” (MIS) or laparoscopic surgery has been rapidly implemented into a variety of surgical disciplines. Accordingly, new risks have emerged and complications of laparoscopic surgery are constantly being evaluated. The Dutch Healthcare Inspectorate conducted a study of the risks presented by MIS procedures and observed many unsubstantiated differences between general surgery, gynecology, and urology. Although the basic knowledge and skills are identical regardless of specialism, multidisciplinary agreements were lacking. The Dutch Healthcare Inspectorate encouraged different specialties performing laparoscopy to work together and develop a multidisciplinary guideline for MIS.

This guideline represents a review of the evidence and consensus clinical opinion. The objective of this guideline is to provide guidance for MIS in daily practice. By formulating multidisciplinary agreements, the aim is to increase patient safety in MIS. It is intended primarily for all specialists performing laparoscopic surgery or those directly involved. This guideline can also be used as a standard by patients, patients’ organizations, hospital organizations, health insurances, and government agencies. The scope of this guideline is laparoscopy in general, specific laparoscopic procedures are not addressed. Different aspects in MIS are described, including laparoscopic entry techniques, pneumoperitoneum, trocar use, electrosurgical techniques, prevention of trocar site herniation, patient positioning, anesthesiology, perioperative care, patient information, multidisciplinary user consultation, and the registration of complications.

In this first of three series papers on the multidisciplinary guideline, we present our literature reviews, conclusions, and practical recommendations for entry techniques and the pneumoperitoneum.

## Methods

The Dutch Society for Endoscopic Surgery together with the Dutch Society for Obstetrics and Gynecology initiated a multidisciplinary working group to develop a guideline on MIS. Two general surgeons, two gynecologists, an urologist, and an anesthesiologist participated in the guideline working group. All were authorized by their scientific professional association (the Dutch Society of Surgery, the Dutch Urological Association, and the Dutch Association of Anesthesiologists, respectively). Because of the surgical technical contents, patients were not involved in the guideline development.

**Fig. 1:**
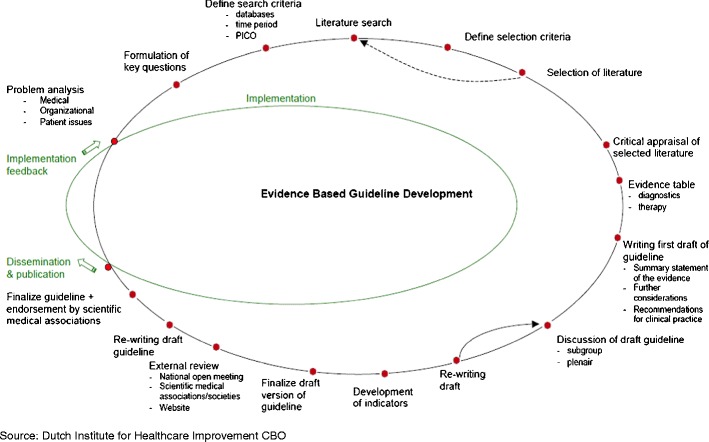
Overview of the developmental process in EBGD

The guideline was developed consistent with the “Appraisal of Guidelines for Research and Evaluation” instrument [1]. Initially, the working group performed a problem analysis to define the scope and topics of the guideline. These problem topics were translated into clinical key questions and the scientific literature was searched for answering the key questions. Separate search strategies were developed for each problem topic. Searches were conducted in collaboration with information specialists. Studies were limited to English and Dutch language in view of the limitations on time and resources. The search strategies are appended (see Appendix). The developers selected relevant literature. The bibliographies of relevant articles were hand searched for other valuable references. The characteristics and methodological quality of the studies were assessed using the checklists from the Dutch Cochrane Center [2]. The evidence was summarized in evidence tables and in the guideline text. The grading system of the Dutch Institute for Healthcare improvement CBO was used to level the evidence (Table 1). The guideline text is structured according to a prescribed Evidence-Based Guideline Development (EBGD) format. Each defined key question has its own text-section that comprises the clinical key question followed by a summary of the literature and a conclusion including the level of evidence. Then, considerations (including: patient preferences, availability of services, organization of care, impact on costs, legal consequences) are discussed and each section ends with recommendations. The recommendations are the practical answer to the key question. They are based on ‘evidence’ (the summary of literature) and balanced with ‘experience’ (the paragraph considerations). An illustrative overview of the EBGD process is shown in Fig. 1.

**Table 1:** Grading system for level of evidence

Level	Studies on therapy/prevention	Studies on diagnostic accuracy	Studies on harm, etiology or prognosis
A1	Systematic review/meta-analysis of at least two independent studies of A2 level with consistent results		
A2	Double-blind randomized controlled trial of good quality and sufficient power	Study with respect to a reference test (gold standard) with pre-defined cut-off values, among large series consecutive persons that received both the index and the reference test and adequate blinding of interpretation of test results	Prospective cohort study of sufficient power and follow-up, adequate control for confounding and selective follow up
			
B	Randomized controlled trial of modest quality or insufficient power, or other analytic study (e.g., case–control study, cohort study)	A comparison with a reference standard that does not meet the criteria required for level A2 evidence	Prospective cohort study that does not meet the criteria required for level A2 evidence. Or retrospective cohort study or case–control study
			
C	Non-analytic study		
			
D	Expert opinion		
			
Level	Conclusion based on		
			
1	One systematic review (A1) or at least two independent randomized controlled trials of level A2		
2	One study of level A2 or at least 2 independent studies of level B		
3	One study of level B or C		
4	Expert opinion		

Grading system used at the Dutch Institute for Healthcare Improvement CBO

## Background

The incidence of laparoscopic complications described in the literature varies considerably. Reported complication rates vary from 1.0 to 12.5 per 1,000, depending on the retrospective or prospective nature of the study, the definition of “complication”, the experience of the surgeons, the characteristics of the study participants, and the complexity of the procedure. The largest reported studies on complication rates in laparoscopy are based on gynecologic procedures. The Finish National Insurance Association registered a total of 256 complications in 70,607 gynecologic laparoscopic procedures (3.6/1,000). The incidence of gastrointestinal injuries was 0.6/1,000, of urological injuries 0.3/1,000 and of vascular injury 0.1/1,000 [3]. A Dutch prospective multicenter study reported 145 complications in a total of 25,764 gynecologic laparoscopies (5.7/1,000). Two fatal cases were described and in 84 procedures a complication resulted in conversion to laparotomy. The incidence of gastrointestinal injuries was 1.13/1,000, and of intra-abdominal vessels 1.05/1,000. Fifty-seven percent of the complications were entry related (closed- and open-entry techniques were included). Women who had undergone a prior history of laparotomy had an increased risk for complications [4].

Other complications in laparoscopy are a pneumothorax or subcutaneous emphysema, with reported incidences of 1.9% and 2.3%, respectively [5]. Pneumothorax is frequently seen together with subcutaneous emphysema and associated with inadequate insufflation of CO_2_ through an improperly placed trocar or Veress needle [6, 7]. Subcutaneous emphysema may also occur when pressurized CO_2_ moves into pre-existing or iatrogenic defects in the diaphragm or retroperitoneum. Due to its good solubility, CO_2_ is rapidly absorbed in the blood circulation and may lead to an increase in hypercapnia and acidosis. Subcutaneous emphysema located in the head and neck area can lead to airway obstruction. The airways should therefore be secured until all emphysema has been dissolved [8].

The incidence of complications related to laparoscopy is low; however, they can be very severe. More than 50% of laparoscopic complications are entry related and these occasionally require emergency surgery. Basically, two different entry techniques can be distinguished׃ the open- and closed-entry technique. Surgeons and urologists often use the open technique with Hasson trocar (also called the Hasson technique) [9] while gynecologists often use the closed technique with blind introduction of the Veress needle and primary trocar. The risks associated with the two different techniques are investigated and can be variously interpreted. The text below describes the strategies for a safe application of entry techniques in laparoscopy.

### Key question

Which entry technique, open or closed, is associated with the lowest risks for complications?

### Summary of the literature

Injuries of the intra-abdominal vessels and bowel are known entry-related complications. Since the incidences of these complications are low, a randomized controlled trial (RCT) would not be the appropriate design to detect risk differences. To detect a risk difference for bowel injury from 0.3% to 0.2%, over 800,000 patients would be needed for inclusion in an RCT [10].

In a Cochrane review, different entry techniques were compared in terms or their influence on intraoperative and postoperative complications [11]. The outcomes were divided into major complications (mortality, vascular injury, bladder injury, bowel injury, gas embolism, and solid organ injury) and minor complications (e.g., extraperitoneal insufflation, trocar site bleeding). Two RCTs were included (a total of 210 patients enrolled) wherein open- and closed-entry techniques were compared. No significant risk differences were found for major and minor complications, neither in more specified analyses. In 2001, the Australian College of Surgeons systematically reviewed the effectiveness and safety of entry techniques for establishing a pneumoperitoneum in laparoscopic surgery. Besides RCTs, other relevant studies with different study designs were included. The outcome data for bowel injury and vascular injury in five nonrandomized prospective and retrospective comparative studies were pooled. A higher risk of bowel injury showed for the open-compared to the closed-entry technique (RR 2.17, 95% CI: 1.14–4.10). No statistical significant risk difference was found comparing the open-versus closed-entry technique for vascular injury (RR 0.68, 95% CI: 0.16–2.84) [12].

Interpreting these results, the potential for selection bias should be taken into account. For example, the open-entry technique would often be the preferred technique in patients with previous abdominal surgery. This selection bias may result in an increased relative risk of bowel injury for the open-entry technique compared to the closed-entry technique.

### Conclusion

**Table:** 

Level 1	No significant risk differences have been found for bowel and vascular injuries, when comparing the open-entry to the closed-entry technique.
	*Evidence level A1* [11]

### Considerations

Theoretically, it could be claimed that retroperitoneal vascular injury can be prevented by using the open-entry technique and thereby eliminating the potential for abrupt and uncontrolled introduction of the primary trocar that may result in a deeper penetration than needed. This risk is inherent in the closed-entry technique and thus vigilance is needed. In practice, it shows that the risk for uncontrolled introduction reduces by gaining experience. No robust conclusions can be drawn from the results of nonrandomized studies because of insufficient power and a high risk of bias.

### Recommendations

- In general, no completely safe entry technique can be recommended. Specialists should preferably practice the technique they have learned and with which they are familiar. According to expert opinion, experience with a particular entry technique will reduce the risks of complications. Exceptions to this rule are: patients with prior abdominal surgery, obese patients, very thin patients and pregnant patients (see key question 1.7).

### Key question

How should the closed- and open-entry techniques be performed?

### Summary of the literature

There are no comparative studies of good methodological quality about differing aspects in specifically closed- and open-entry techniques. The majority of the studies are descriptive and based on expert opinions.

## Closed-entry technique: placement of the Veress needle

The Veress needle will be checked for its potency and spring action before inserting it into the abdomen. This is to ensure a free flow of CO_2_ and the protective function of the blunt tip. The blunt tip emerges out of the sharp end to protect the bowel and other intra-abdominal organs from inadvertent puncture.

The abdominal wall is lifted until a 45° angle to the horizontal. This can be done by lifting the skin at the umbilicus by hand or with a towel clip. In the Cochrane review on laparoscopic entry techniques, one RCT was included comparing abdominal wall lifting versus not lifting for placement of the Veress needle. Not lifting the abdominal wall showed less failed entries, with no difference in complication rate. According to the experts however, abdominal wall lifting is aimed to prevent compression and thereby reduction of distance between instruments and retroperitoneal structures. The Veress needle is inserted perpendicular to the fascia and then directed towards the surgical field, avoiding the major vessels.

There are several tests to verify the correct position of the Veress needle tip. Different tests were evaluated in an observational study and it was concluded that a low initial gas pressure (<10 mmHg) followed by a free influx of CO_2_ is the only valuable measure to reflect correct intraperitoneal Veress needle placement [13].

### Conclusions

**Table:** 

Level 3	A low initial gas pressure (<10 mmHg), followed by a free influx of CO_2_, is a reliable indicator of correct intraperitoneal Veress needle placement
	*Evidence level B* [13]
Level 4	There are insufficient high-quality comparative studies on safety and effectiveness of the different aspects in the specific open- and closed-entry techniques
	*Evidence level D* (*opinion of the guideline development group*)

### Considerations

In practice, for selected patients, only specific entry techniques are applied. An adequate selection is required, which is discussed among key question 1.7.

During every laparoscopic procedure, instruments to perform a laparotomy should be available. These could be necessary in the event that an injury occurs for which a conversion is required. Prior to the closed-entry technique, the patient is catheterized or an indwelling catheter is inserted. A nasogastric tube can be used. A filled stomach or bladder may hinder the placement of laparoscopic instruments or these structures can be damaged. Positioning the patient in Trendelenburg prior to the insertion of laparoscopic instruments could theoretically increase the risk for inadvertent aortic puncture. In most cases, the primary incision is preferred in the umbilicus because it overlies the location where the skin, fascia, and parietal peritoneum converge and fuse. Consequently, the distance between skin and abdominal cavity is short and an umbilical incision generally has a good cosmetic result. The skin incision should be large enough to prevent overshoot injury. In lean patients, initial skin incision should not involve the fascia. One should avoid stab incision. These precautions are not sufficiently investigated, but are considered common sense.

## Closed-entry technique: insertion of the primary trocar

After the pneumoperitoneum is achieved and the Veress needle is removed, the primary trocar is inserted through the umbilical incision in the same directions as the Veress needle. When using a normal intra-abdominal pressure (IAP; 12–16 mmHg), the umbilicus should be lifted and fixated as with the insertion of the Veress needle. Every move associated with introduction of the instrument should be well controlled. It is recommended to open the valve of the trocar to hear if the tip is located in the abdominal cavity. After inserting the laparoscope, visual inspection is intended to check for iatrogenic injuries and intraperitoneal aberrations.

## Open-entry technique: insertion of the primary trocar

In the open-entry technique, the introduction of sharp instruments is avoided. A small incision is created and the layers of the abdominal wall are incised. The peritoneum is opened bluntly or sharply. When reaching the peritoneal cavity this is often visible and can be verified by palpation with a finger. The primary trocar is then inserted and CO_2_ is inflated to create the pneumoperitoneum. Today, balloon blunt-tip trocars are commonly used. The distal end of the sleeve has an inflatable balloon to create an air-tight fixation of the trocar.

## Trocar removal

At the end of each laparoscopic procedure, the removal of all trocars should be under direct vision. As yet, unnoticed injuries, e.g., tamponaded hemorrhage or bowel perforation, can be detected. Thereby, trocar site herniation can possibly be prevented by avoiding bowel or omental tissue is pulled into the trocar site.

### Recommendations for the closed-entry technique

- Prior to continuing the insufflation, the initial IAP should be <10 mmHg (measured via Veress needle).
- Prior to the closed-entry technique, it is preferable to insert a nasogastric tube and to empty the urinary bladder.

### General recommendations for primary entry

- Instruments to perform an emergency laparotomy should be available at close hand. These could be necessary in the event that a complication occurs for which conversion is required.
- During primary entry, the patient must be positioned horizontally until the primary trocar is safely inserted. The umbilicus can be stabilized by lifting it this can prevent compression and consequent reduction of the distance between the instruments and retroperitoneal structures.
- After opening the peritoneum and prior to the introduction of the (blunt) primary trocar, it is important to ensure that the peritoneal cavity has been reached.
- The primary trocar must be introduced in a controlled manner, at an angle of 90° to the fascia. Once the peritoneal cavity has been reached, the insertion must be stopped immediately.
- After introduction of the laparoscope, the abdomen must be inspected for adjacent bowel by rotating the laparoscope 360°. If adjacent bowel is observed, it must be inspected for (signs of) hemorrhage, lesion, or retroperitoneal hematoma.
- The removal of all trocars should under direct vision, to recognize a tamponaded hemorrhage or a bowel perforation that has not been noticed, and to prevent bowel or omental tissue being pulled into the trocar site.

### Key questions

Closed-entry technique and IAP:

1. What IAP should be achieved prior to insertion of the primary trocar?
2. What IAP should be applied once the insertion of trocars is complete?

### Summary of the literature

When applying “peritoneal hyperdistention”, the abdomen is insufflated to 25–30 mmHg before inserting the primary trocar. After introduction of the trocars, the IAP is reduced to a normal pressure (12–16 mmHg).

Prospective observational studies have shown that the increased size of the “gas bubble” has a splinting effect and allows the trocar to be more easily inserted through the layers of the abdominal wall. Furthermore, when force is applied to a hyperdistended abdomen (25 mmHg), the depth under the umbilicus is larger, compared to a normally distended abdomen (10 mmHg) [14]. An increased IAP induces a hemodynamic stress response. The venous return from the lower extremities alters, cardiac output decreases and there is an increase in mean arterial pressure (MAP) systemic, pulmonary and vascular resistances [15–17]. In a prospective observational study, significant hemodynamic changes were observed when the IAP was elevated above 12 mmHg. There was a decrease in stroke volume and cardiac output and an increase in MAP and systemic vascular resistance [18].

No studies were found that analyzed for the upper limit of IAP. Neither systematic reviews nor RCTs evaluating the clinically relevant consequences of “peritoneal hyperdistention” were found. In a prospective cohort study including 100 women undergoing gynecological laparoscopy, hemodynamic changes were analyzed. High pressures (25–30 mmHg) resulted in minimal changes in heart rate and blood pressure and a statistical significant decrease of pulmonary compliance, all without clinically relevant consequences [19]. This study was conducted in healthy women with classified American Society of Anesthesiologists scores (ASA) I and II. The hemodynamic and pulmonary consequences of “peritoneal hyperdistention” has not been studied in men and patients with higher ASA scores. A larger prospective cohort study (1,150 consecutive ASA I patients undergoing gynecological laparoscopy) investigated the safety of the pressure technique for insertion of the primary trocar. No insertion complications or adverse clinical effects were noted during hospital stay [20].

In a Cochrane review, the harms and benefits of the low pressure pneumoperitoneum (<12 mmHg) compared with standard pressure pneumoperitoneum (12–16 mmHg) were assessed in patients undergoing laparoscopic cholecystectomy [21]. A total of 15 RCTs were included (690 patients), all with high risk of bias. There was no difference in mortality, postoperative complications, or conversion to open cholecystectomy between the groups. None of the trials reported any cardiopulmonary complications. Only patients with ASA I scores were included in the trials, together with a low overall incidence of cardiopulmonary complications (0.5% in a case series of 400 patients, 70% of the patients were scored ASA I) [22] the meta-analysis was under powered. In seven trials, the outcome data were incomplete: reasons for conversion were not reported. This caused a high risk of bias and thus the safety of the low-pressure pneumoperitoneum could not be ascertained [21].

### Conclusions

**Table:** 

Level 1	The safety of low pressure pneumoperitoneum (<12 mmHg) has only been studied in patients undergoing cholecystectomy. It is uncertain whether low pressures in comparison with conventional pressures, result in equal risks of morbidity and conversion to open surgery.
	*Evidence level A1* [21]
Level 2	Elevated IAP above 12 mmHg is associated with significant hemodynamic effects. These effects did not demonstrate any clinically relevant consequences
	*Evidence level A2* [18]
Level 3	“Peritoneal hyperdistention” has only been studied and found to be safe in healthy female patients with ASA scores I or II
	*Evidence level C* [19, 20]
Level 3	“Peritoneal hyperdistention” (insufflation to IAP 25–30 mmHg), results in an increased size or “gas bubble” and a splinting effect of the abdominal wall, compared to the traditional, limited-volume pneumoperitoneum
	*Evidence level C* [14]

### Considerations

The abdominal wall cannot be lifted when the abdomen is hyperdistended. Thus when using the “peritoneal hyperdistention” technique, the primary trocar is inserted perpendicular to the abdominal wall.

“Peritoneal hyperdistention” can result in hemodynamic changes and compromise the respiratory ventilation of the patient. The anesthesiologist should therefore be informed when changing the IAP. “Peritoneal hyperdistention” should last no longer than necessary: after introduction of the trocars, the IAP should be reduced to a normal pressure (12–16 mmHg). High pressures did not result in any clinical relevant compromises in healthy patients but could possibly have more clinically significant effects in patients with ASA III and IV scores.

RCTs have shown that the use of a low pressure pneumoperitoneum results in less hemodynamic changes [22], less shoulder pain [23, 24], less postoperative pain [25], and less use of analgesics [24, 25]. However, main criticism of low pressure pneumoperitoneum is its ability to provide adequate surgical exposure and its safety.

### Recommendations for IAP

- Before blind introduction of the primary trocar, the IAP must be at least 12–16 mmHg. The “pressure technique” to 25–30 mmHg may be applied briefly in selected patients.
- After introduction of the trocars, the IAP must be reduced to a normal pressure (12–16 mmHg, depending on patient characteristics) creating sufficient distension to perform laparoscopy and where the anesthesiologist can provide safe and effective pulmonary ventilation.

### Key question

What alternative entry techniques are available?

### Summary of the literature

#### Direct trocar entry

The direct trocar entry has been described as an alternative to the Veress needle technique. The primary entry is initiated with one blind step instead of two (Veress needle and trocar). The direct trocar entry is faster than any other method of entry [26]. In the Cochrane review on laparoscopic entry techniques, a meta-analysis was performed comparing direct trocar entry to Veress needle entry. A total of 1,909 participants in six RCTs were included and no major complications occurred with both techniques [27–32]. There were however, statistically significant reductions in the risk of extraperitoneal insufflation and failed entry in the direct-entry group (OR 0.06; 95% CI: 0.02–0.023 and 0.22; 95% CI: 0.08–0.56, respectively) [11].

#### Other entry systems

Different entry-systems have been developed to reduce the risk for entry-related complications: direct-vision entry systems [33, 34], radially expanding trocars [35], tapered blunt tipped trocars (TrocDoc, second generation Endotip®) [36]. For the Cochrane review, no RCTs comparing direct-vision versus Veress needle entry were identified. There were no other observational studies with sufficient power to demonstrate a risk reduction for major complications when direct-vision entry was used. The Cochrane review on laparoscopic entry techniques concludes that radially expanding access trocars offer advantages in terms of reduced trocar site bleedings, less extraperitoneal insufflations, and failed entries [11, 34, 37, 38]. RCTs and other observational studies comparing tapered blunt tip systems with the conventional Veress needle or open-entry technique did have insufficient power to demonstrate risk reductions for any complication.

Needlescopes are optical Veress needles with 1–2 mm diameter [39]. There is as yet no evidence for their superiority compared to the conventional Veress needle entry.

### Conclusions

**Table:** 

Level 1	Direct trocar entry leads to fewer extraperitoneal insufflations and failed entries when compared with Veress needle entry
	*Evidence level A1* [11, 27–32]
Level 1	For primary entry, radially expanding access trocars reduce the risks for trocar site bleedings, extraperitoneal insufflations and failed entries compared to conventional trocars
	*Evidence level A2* [11, 34, 37, 38]
Level 3	There is no evidence that use of direct vision systems, a tapered blunt tipped trocar or a needlescope for primary entry is safer than the conventional open- or closed-entry techniques
	*Evidence level C* [26, 40]

### Considerations

Some RCTs excluded specific patient groups, e.g., patients with previous abdominal surgery, obese patients, or patients at risk for sub-umbilical adhesions. Therefore, the results of these RCTs do not apply for the complete laparoscopic patient population. For a select patient group, the direct trocar entry seems a safe and fast method with a lower risk for extraperitoneal insufflation and failed entry compared to Veress needle entry. This technique is not widely used in laparoscopic practice, probably because extensive experience is required for its use.

Moreover, high costs of newly developed systems could be a limiting factor for their use. Studies on cost effectiveness should be conducted to make informed choices for the use of specific instruments in laparoscopic practice.

### Recommendations for alternative entry techniques

- The guideline development group does not recommend the use of direct trocar entry, since a high level of experience is needed for the safe application of this technique.
- The use of visual entry systems is only recommended when an adequate pneumoperitoneum (with the Veress needle) has been created.
- Radially expanding trocars are an expensive alternative to standard trocars. The use of these trocars may reduce trocar site bleedings and extraperitoneal insufflations.

### Key question

What alternative sites can be safely used for insertion of the Veress needle and primary trocar?

### Summary of the literature

The rate of adhesion formation at the umbilicus may occur up to 50% in patients following midline laparotomy and 23% following low transverse incision [41]. A Veress needle or trocar should never be blindly inserted at a site where adhesions may be expected. In those cases, the umbilicus is not the appropriate site for closed-entry. The most usual alternative site following laparotomy is in the left upper quadrant via Palmer’s point. Palmer’s point is located 3 cm below the costal margin in the midclavicular line. Adhesions are rarely formed in this area, though, in cases of previous surgery in this area or splenomegaly, Palmer’s point may as well be inappropriate.

It remains unclear what rates of adhesion formation are found in patients following laparoscopy and thus which entry location is most suitable following a prior laparoscopy.

### Conclusion

**Table:** 

Level 3	When periumbilical adhesions may be expected, Palmer’s point is the appropriate site for insertion of the Veress needle and primary trocar
	*Evidence level C* [41]

### Considerations

When periumbilical adhesions are suspected, either an open entry technique or a closed entry at a different location (preferably Palmers’ point) must be performed. It could be an option to insert the primary trocar sub-umbilical, after first having excluded periumbilical adhesions with a needlescoop [42].

Other sites for insertion of the Veress needle and trocar have been described (suprapubic, through the uterine fundus or posterior fornix) but, given the greater risks of complications, are to be avoided.

### Recommendation for alternative entry site

- In the event of doubt or suspected periumbilical adhesions, the Veress needle and primary trocar should not be introduced at the umbilicus. An alternative technique (e.g., the open entry technique or insufflation at the point of Palmer) should be chosen.

### Key question

How should secondary ports be created?

### Summary of the literature

The safety of different methods to create secondary ports has not systematically been studied. A prospective observational study showed that 64% of the superficial epigastric vessels could be identified with transillumination. Laparoscopic visualization successfully identified 82% of the inferior epigastric vessels. Both methods were less effective as patient’s weight increased [43].

The insertion of secondary ports should be visualized laparoscopically, taking care to avoid injury to the vessels and viscera. Suprapubic insertion of a trocar puts the bladder at risk of damage; therefore, the bladder should be visualized. If the margins of the bladder are unclear, the bladder can be filled retrograde.

### Conclusion

**Table:** 

Level 3	Superficial epicastric vessels can be visualized with transillumination. Deeper epigastric vessels can be visualized laparoscopically
	*Evidence level B* [43]

### Considerations

Secondary ports are inserted perpendicular to the skin to minimize the iatrogenic defect in the fascia. Once the tip has passed the peritoneum, it is directed towards the surgical field. The inferior epigastric vessels should be visualized laparoscopically to ensure that the entry site is away from the vessels. The deep epigastric arteries and venae comitantes are located lateral to the lateral umbilical ligaments. The visualization can be difficult in obese patients. Then, the incision should be placed lateral to the rectus sheath, taking care to avoid injury of the pelvic side wall.

### Recommendations for secondary ports

- The superficial epigastric vessels should be visualized by translumination prior to the insertion of secondary trocars. Deeper epigastric vessels should be visualized laparoscopically.
- When inserting the secondary trocars, this must be under direct vision and with the presence of an adequate pneumoperitoneum. The trocars should be inserted perpendicular to the fascia and then directed towards the surgical site.
- When a suprapubic port is inserted, attention must be paid to the localization of the bladder. Retrograde filling of the bladder is possible.

### Key question

What entry techniques should be applied for laparoscopy in a pregnant patient, a patient who is very thin or a patient with morbid obesity?

### Summary of the literature

No trials that compare different entry techniques in pregnant patients or very thin patients or patients with morbid obesity have been described. There is some descriptive literature for these specific patient groups.

## The pregnant patient

Concerning pregnant patients, there are specific concerns for a higher likelihood of injury to the uterus or other intra-abdominal organs. From 12 weeks of gestation, the fundal height of the uterus increases rapidly. The Society of American Gastrointestinal and Endoscopic Surgeons (SAGES) published a guideline on laparoscopy in pregnancy [44, 45]. In this guideline, the authors recommend that in the second and third trimesters of pregnancy, the site of entry should be adapted to the fundal height: from the umbilicus towards subcostal regions [46]. In their opinion, through this adjustment together with elevation of the abdominal wall during insertion, both the Hasson technique and Veress needle entry could be safely and effectively utilized.

## The very thin patient

In children and extremely thin patients (BMI <18 kg/m^2^), the aorta may lie less than 2.5 cm under the skin [47]. These patients are at particular risk for retroperitoneal vascular injury during primary entry and for this reason, the open entry technique or closed entry at Palmer’s point are preferable

## The obese patient

The site for primary entry, umbilicus or Palmer’s point, in obese patients should depend on the body habitus and distribution of fat. The location where the thinnest subcutis is expected is best used for inserting the Veress needle or trocar. The open as well as the closed-entry technique can be applied. If the Veress needle is inserted vertically downward at the umbilicus, the mean distance from the lower margin of the umbilicus to the peritoneum is 6 cm (with a standard deviation of 3 cm). In this way, it is possible to use a Veress needle with standard length, even in extremely obese patients.

### Conclusion

**Table:** 

Level 4	There is insufficient qualitative data comparing the safety of different entry techniques in pregnant patients, very thin patients and patients with morbid obesity
	*Evidence level D* (*opinion of the MIS guideline development group*)

### Considerations

In a pregnant patient, blind insertion of a Veress needle or trocar gives an additional risk for injury of the uterus. The SAGES describes a closed entry can be considered, however, in our opinion, an open-entry technique is preferable. Since the fundal height can be increased in the first trimester due to a twin pregnancy or myomas, the open techniques is recommended in all trimesters. Attention should be paid to other aspects of laparoscopy in pregnancy as well: positioning, IAP, fetal monitoring, and possibly medicinal tocolysis.

### Recommendations for specific patient groups

- In pregnant patients, the open-entry technique or closed-entry technique via Palmers’ point is preferred.
- In underweight patients (BMI <18 kg/m^2^ and children), the open-entry or closed-entry technique via Palmers’ point is preferred.
- In patients with morbid obesity (BMI >40 kg/m^2^), the closed-entry technique via the umbilicus or Palmers’ point is preferred.

## Appendix

**Table 2:** Literature search for entry techniques

Subject	Database	Search terms
Entry	Medline (OVID) 1950-Aug.2010	1. Laparoscopy/
		2. exp *Laparoscopy/
		3. Surgical Procedures, Minimally Invasive/
		4. “lapar*scop*”.m_titl.
		5. “minimal invasive*”.m_titl.
		6. or/1–5
		7. (“laparoscopic injur*” or “laparoscopic entr*” or “laparoscopic adj2 complication*” or “closed laparoscop*” or “open laparoscop*” or “direct-entry adj2 laparoscop*”).ti,ab.
		8. 6 and 7
		9. limit 8 to yr = “2006 -Current”
		10. RCT (filter)
		11. SR (filter)
		12. exp epidemiological studies/
	Embase	laparoscop*:ti OR ‘laparoscopy’/exp/mj OR ‘minimal invasive’:ti OR ‘laparoscopic surgery’/exp AND ((laparoscopic NEAR/1 injur*):ab,ti OR (laparoscopic NEAR/1 entr*):ab,ti OR (laparoscopic NEAR/1 complication*):ab,ti OR (closed NEAR/1 laparoscop*):ab,ti OR (open NEAR/1 laparoscop*):ab,ti OR ‘direct entry’:ab,ti) AND [embase]/lim AND [2006–2011]/py
Pneumo-peritoneum	Medline (OVID) 1950-March 2010	1. exp *Laparoscopy/
		2. Surgical Procedures, Minimally Invasive/
		3. “lapar*scop*”.m_titl.
		4. “minimal invasive*”.m_titl.
		5. or/1–5
		6. Pneumoperitoneum, Artificial/ae [Adverse Effects]
		7. 6 and 7
	Embase	laparoscop*:ti OR ‘laparoscopy’/exp/mj OR ‘minimal invasive’:ti OR ‘laparoscopic surgery’/exp AND (intraperitoneal NEAR/5 pressure OR intraperitoneal NEAR/5 insufflation) NOT [animals]/lim)
		Searchfilter RCTs

The literature search for entry techniques was based on the search strategy of the Green-top Guideline “Preventing entry-related gynecological laparoscopic injuries” from the Royal College of Obstetricians and Gynaecologists. Medline and Embase were searched for relevant randomized controlled trials, systematic reviews and meta-analyses. The search was restricted to articles published in Dutch and English from 1966 to Augustus 2010
